# Outcomes of Refractive Surgery Consultations at an Academic Center: Characteristics Associated with Proceeding (or Not Proceeding) with Surgery

**DOI:** 10.1155/2020/4354085

**Published:** 2020-03-30

**Authors:** Irene C. Kuo, Benjamin Lee, Jiangxia Wang

**Affiliations:** ^1^Wilmer Eye Institute, Department of Ophthalmology, Johns Hopkins University School of Medicine, Baltimore, MD, USA; ^2^Meharry School of Medicine, Nashville, TN, USA; ^3^Department of Ophthalmology, Louisiana State University, New Orleans, LA, USA; ^4^Johns Hopkins Biostatistics Center, Bloomberg School of Public Health, Baltimore, MD, USA

## Abstract

**Objective:**

Refractive surgery volume has not rebounded despite economic recovery and literature describing safety, efficacy, and high patient satisfaction. We sought to examine characteristics of consultation seekers and status after consultation.

**Methods:**

Charts of patients seeking refractive surgery at Johns Hopkins University from 2013 through 2016 were retrospectively reviewed for age, gender, refractive characteristics, and outcome: surgery (photorefractive keratectomy, laser in-situ keratomileusis, implantable collamer lens, or refractive lens exchange); no surgery—“lost candidate” (good candidates who were lost after consultation); noncandidates based on technological limitations or contraindications; or no surgery—possessing expectations that surgery would not meet. Associations between characteristics and status after consultation were examined.

**Results:**

Twenty percent (142/712) of all patients were “lost candidates”; 57% (408/712) completed surgery. More women (56% or 401/712) sought consultation, but a greater percentage (63% or 195/311) of men completed surgery than women did (53% or 213/401) (*p*=0.02). Of consultation seekers, 60% were low myopes, 29% were high myopes (>6 diopters of myopic spherical equivalent), and 11% were hyperopes. Surgical patients' mean age was 34.2 ± 10.2 (standard deviation) years; for each additional year of age, patients were less likely to have surgery (*p* < 0.001). Hyperopes were ≥3 times more likely than myopes to have expectations not met by surgery or to be noncandidates than to have surgery (*p* < 0.005).

**Conclusions:**

Most patients seeking refractive surgery had 6 diopters or less of myopia. About 20% of patients were lost after consultation; better counseling and follow-up of candidates may be warranted. Expectations and technology limit eligibility for many, especially hyperopes. Low surgery volume may affect training of future refractive surgeons.

## 1. Introduction

In recent years, the volume of refractive surgery (notably photorefractive keratectomy (PRK) and laser in-situ keratomileusis (LASIK)) has dropped from approximately 1.4 million a year in the United States in the late 1990s-early 2000s to currently 650,000 a year despite documented outcomes of improved quality of life and high patient satisfaction worldwide, comparable to or exceeding other elective procedures [1–3]. By some estimates, under 1% of adults with refractive error in the US undergo refractive surgery each year, implying many more adults are eligible for surgery.

While the 2008 economic recession probably had an impact, other factors, such as demographic changes and increased electronic device use, may be contributing to low volume and lack of rebound with economic recovery. In the time since a publication on trends in refractive procedures [4], small incision lenticule extraction (SMILE) and implantable collamer lenses (ICLs) have been adopted and would be expected to increase interest in refractive surgery over all.

The purpose of this study was to analyze characteristics of individuals seeking refractive surgery consultation at an academic institution in order to understand patient wants and needs, to ascertain characteristics of patients who proceed (or do not proceed) to surgery, and to understand where our evaluations or procedures fall short.

## 2. Materials and Methods

The Institutional Review Board of Johns Hopkins University granted approval for the protocol under which this chart review was conducted (IRB00094392). This review was conducted in accordance with the tenets of the Declaration of Helsinki.

The Wilmer Eye Institute of Johns Hopkins University maintains a record of candidates seeking refractive surgery consultation at the satellite clinic housing the laser suite. All candidates undergo a thorough preoperative evaluation, including medical, ophthalmic, and social history; preoperative uncorrected and best spectacle-corrected distance and near visual acuities; Scheimpflug imaging (Pentacam; Oculus, Arlington, WA); biomicroscopic and dilated examinations; pupillary examination; Schirmer testing; pachymetry; and manifest and cycloplegic refractions. Manifest refraction of myopic candidates is aimed at the least minus refraction to see the most Snellen letters; hyperopic patients are pushed with the most hyperopic refraction, often confirmed with cycloplegic refraction and/or relaxation of accommodation over time wearing an updated prescription glass in cases of latent hyperopia. In all situations, manifest refraction, cycloplegic refraction, and wavefront refraction are compared and used to determine the eventual refractive treatment. The option of glasses and/or contact lenses is always discussed. All PRK and LASIK were wavefront-guided using the VISX Star S4 CustomVue (Johnson and Johnson Vision, Santa Ana, CA) with eye tracking and iris registration where possible. Patients seeking presbyopia-correcting lens implants were not included in this study; presbyopic inlays are not performed at our institution.

Pertinent characteristics of patients seen between January 1, 2013, and December 31, 2016, were age, gender, manifest refraction, and status after consultation; study inclusion required adequate documentation of the above. Refractive error was categorized by the mean spherical equivalent of the manifest refraction (MRSE) of a patient's two eyes: “low myopia” for 6 diopters (D) of myopia or less, “high myopia” for >6 D of myopia, and “hyperopia” for any hyperopic error.

There were four status categories: (1) surgery, (2) no surgery—“lost candidate” (good candidates who were lost after consultation), (3) no surgery—expectations would not be met, and (4) noncandidate. Surgery type (PRK, LASIK, ICL, and refractive lens exchange) and correction (full vs. monovision variant) were recorded. The four categories were chosen prior to chart analysis. An individual was a “lost candidate” if he or she was deemed a good surgical candidate and did not express concerns about cost, risk, or other factors, yet did not proceed to surgery. Subcategories were developed retrospectively as certain patterns emerged after review of the first few hundreds of charts. “Expectations would not be met” included “seeking presbyopic correction” and “others” (e.g., patient expressed the cost of surgery exceeded perceived benefit). “Noncandidate” consisted of “ophthalmic contraindication” (e.g., ectasia risk), “technological limitation” (e.g., high hyperopia or corneal astigmatism), and “not treatable by refractive surgery” (e.g., cataract, amblyopia). At this site, most consultations are performed by optometrists who refer suitable candidates to the surgeon. The refractive coordinator attempts to arrange a brief meeting with the refractive surgery chief the same day. Otherwise, another surgeon is found, or the patient is asked to meet a surgeon another day.

For the univariate analysis, patient characteristics were compared by status after consultation. Patient age was compared using one-way analysis of variance (ANOVA). Chi-square test was used to determine whether gender and refractive categories were related to status after consultation. Multinomial logistic regression was performed to examine associations between patient characteristics (refractive category, gender, and age) and status after consultation; relative risk ratios (RRRs) with 95% confidence intervals (CIs) were presented. Potential interaction terms among age, gender, and refractive categories were examined for associations. In analyses not involving exact age, patients were grouped by 21–39, 40–59, ≥60 years of age to reflect onset of presbyopia, and other age-related eye conditions. A *p* value ≤0.05 was considered statistically significant for all analyses. All data were analyzed using STATA software version 14.2 (StatCorp, College Station, TX). 

## 3. Results

Of the 868 patient charts reviewed, 712 (82%) met the inclusion criteria and were included in the analysis. The main reason for exclusion was lack of electronic charting (adopted in 2013) and lack of access to paper charts.

A higher proportion of consultation seekers were female than male (*p*=0.012) (Table 1). The majority of consultation seekers were 21–39 years of age (*p*=0.001). Young women formed the largest of the 6 age- and gender-based subcategories. The smallest category was men ≥60 years.

Hyperopic patients (mean age: 51.0 ± 12.4 years) were about 15 years older than high myopes (34.8 ± 10.0 years) or low myopes (36.7 ± 11.6 years) (*p*=0.001). Of consultation seekers, 425 (60%) were low myopes (mean MRSE ± standard deviation (SD): −3.64 ± 1.50 D), 203 (29%) were high myopes (−7.72 ± 0.86 D), and 81 (11%) were hyperopes (+2.55 ± 0.55 D). Thirteen patients had one eye that would be characterized as low myopia and the other eye as high myopia. Because the mean of both eyes' spherical equivalent was used to classify patients as low myopia, high myopia, and hyperopia, 5 such patients were characterized as high myopia, and 8 patients were characterized as low myopia. The mean difference in MRSE between patients' two eyes was 0.06 ± 1.32 D.

### 3.1. Types of Surgery

**Figure 1:**
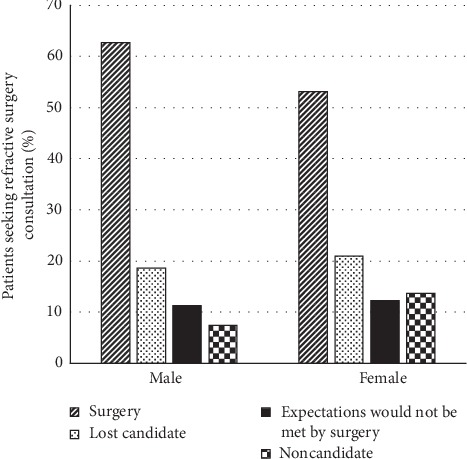
Outcomes after refractive surgery consultation relative to gender.

**Figure 2:**
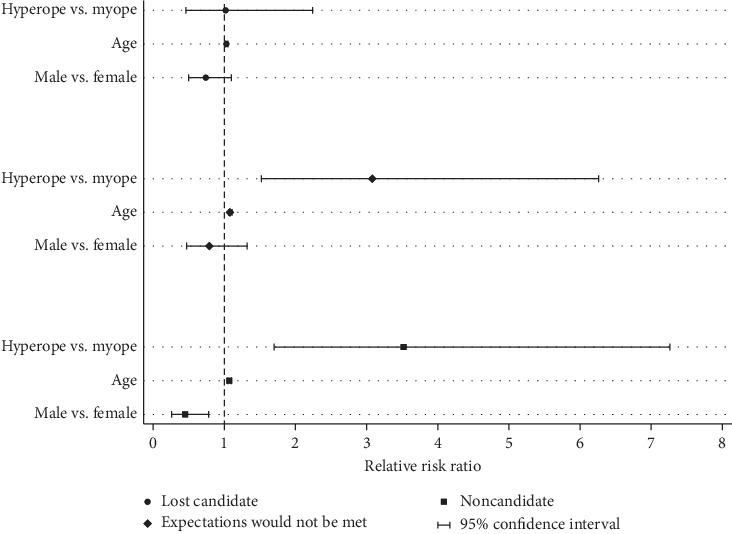
Multinomial logistic regression estimates displaying relative risk ratios of various outcomes relative to surgery for categories of refractive error, age, and gender.

Of consultation seekers, 57% (408/712) completed refractive surgery; 49% (201/408) underwent PRK, 42% (170/408) had LASIK, 8% (35/408) underwent ICL implantation, and 0.5% (2/408) underwent refractive lens exchange. Ninety-one percent (370/408) opted for full correction; the remainder had monovision or mini-monovision. Most charts did not document discussion of monovision trial with contact lenses or trial frames. A greater percentage (63% or 195/311) of men who sought consultation completed surgery than women did (53% or 213/401) (*p*=0.02; Figure 1). Men were less likely than women to be noncandidates than to have surgery (RRR = 0.44; 95% CI [0.25, 0.77]; *p*=0.004) (i.e., men were 0.44 times as likely as women to be noncandidates than to undergo surgery) (Figure 2). No gender difference existed in categories of lost candidate or having expectations not met by surgery.

### 3.2. Outcomes of Consultation

**Figure 3:**
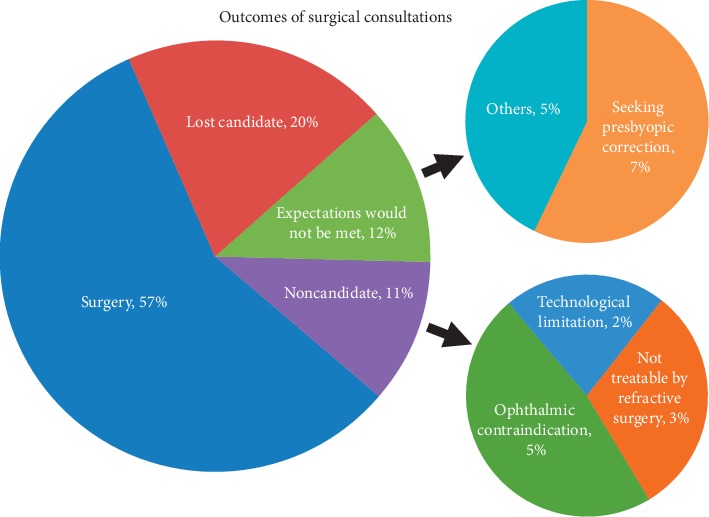
Outcomes of surgical consultation with breakdown of patients not proceeding to surgery because of expectations or technological, medical, ophthalmological, or other limitations.

The proportions of consultation seekers by category are shown in Figure 3, with 57% of 712 patients proceeding to surgery, 20% being lost candidates, and 12% having expectations that would not be met by surgery. The remaining 11% (78/712) of patients interested in surgery were categorized as noncandidates from a medical/ophthalmic contraindication (5.2% or 37/712; 47% of noncandidates), technological limitation (2.4% or 17/712; 22% of noncandidates), or condition not treatable by refractive surgery (3.3% or 24/712; 31% of noncandidates). Leading ophthalmic contraindications were ectasia/thin cornea (10/78 or 13% of all noncandidates) and dry eye (13%), followed by anatomical considerations for ICL (3/78 or 4%), lack of stable refractions (2), keratoconus (2), pellucid marginal degeneration (2), anterior basement membrane dystrophy (2), and Fuchs corneal dystrophy (1). Medical contraindications (4% of all noncandidates) included poorly controlled diabetes (2) and autoimmune disease (2). Technical limitations were led by high corneal astigmatism (10/78 or 13% of all noncandidates) and hyperopia exceeding limits of the laser platform (5/78 or 6.4%), followed by irregular topography (2). Cataract topped conditions not treated by refractive surgery (17/78 or 22% of all noncandidates), followed by small numbers of amblyopia (3), corneal scar (2), poor vision following retinal detachment (1), and failed ICL (1). Patients with cataracts were referred appropriately.

### 3.3. Outcomes by Age

**Figure 4:**
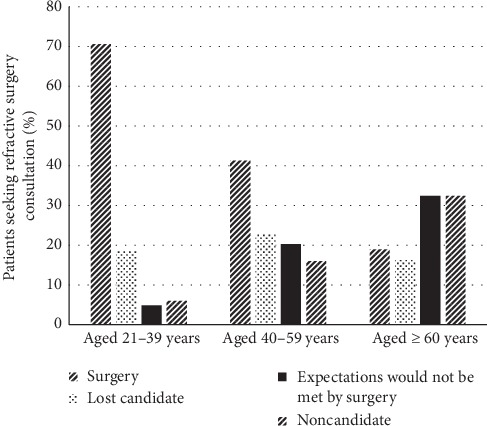
Outcomes of refractive surgery consultation relative to age group.

Seventy percent (295/418) of consultation seekers in 21–39 years of age underwent surgery (Figure 4). The mean ages were as follows: surgical patients—34.2 ± 10.1 years; lost candidate—38.3 ± 12.0 years; patients whose expectations would not be met—47.4 ± 12.1 years; noncandidates—45.8 ± 14.4 years (ANOVA, *p* < 0.001). Fewer than 20% (7/37) of candidates ≥60 years of age underwent surgery; 65% (24/37) either had expectations that would not be met or were noncandidates. Compared with undergoing surgery, older patients were more likely to be lost candidates (RRR: 1.04; 95% CI [1.02, 1.04]; *p* < 0.001) (i.e., for each additional year of age, patients on average were 1.04 times more likely to be in this category than to undergo surgery); to have expectations that would not be met (RRR: 1.08; 95% CI [1.06, 1.11]; *p* < 0.001); or to be noncandidates (RRR: 1.07; 95% CI [1.05, 1.09]; *p* < 0.001) (Figure 2).

### 3.4. Outcomes by Refractive Error

**Figure 5:**
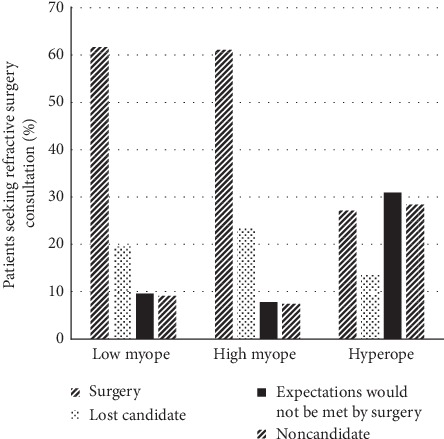
Outcomes of refractive surgery consultation relative to refractive error.

Approximately 60% (386/628) of all myopes proceeded to surgery, in contrast with 27% (22/81) of hyperopes (Figure 5). Twenty-one percent (131/628) of all myopes and 14% (11/81) of hyperopes were lost candidates despite good surgical candidacy. About a third (25/81) of hyperopic patients had expectations that would not be met, and about a third (23/81) were noncandidates (Figure 5). Compared with having surgery, hyperopic patients were >3.5 times more likely than myopic patients to be noncandidates (RRR: 3.52; 95% CI [1.70, 7.26]; *p*=0.001) and 3 times more likely to have expectations that surgery would not meet (RRR: 3.08; 95% CI [1.52, 6.26]; *p*=0.002) (Figure 2).

Of patients who had surgery, 64% (262/408) were low myopes, 30% (124/408) were high myopes, and 5% (22/408) were hyperopes (*p* < 0.001). Hyperopes formed the smallest proportion (11/142 or 8%) and low myopes the largest proportion (83/142 or 58%) of lost candidates (*p* < 0.001). Interaction terms between age and gender or between refractive error category and gender were not significant.

The median number of days between refractive surgery evaluation to LASIK or PRK was 29 days (75^th^ percentile: 62 days). For ICLs, it was 56 days (75^th^ percentile: 110 days). The time between evaluation and refractive lens exchange in the right and left eyes of one patient was 192 and 206 days, respectively.

## 4. Discussion

The downturn in refractive surgery despite high patient satisfaction [1–3] warrants examination of possible factors. We noted some patient characteristics and consultation outcomes, such as low myopes, forming the largest group of seeking refractive surgery yet 20% being good candidates who did not proceed to surgery and young men being the most likely to complete surgery although young women were the largest group seeking consultation. Elucidation of such characteristics and outcomes may help improve consultation quality. Although other papers have investigated reasons for surgeons not performing LASIK in certain candidates [5–7], our paper describes nonsurgical patients in terms of other than medical contraindications (e.g., keratoconus, high myopia, high astigmatism, and thin corneas) [5–7] and ascribes some consultation outcomes partly to patient, optometrist, and surgeon behavior/choice. As a substantial percentage of good candidates was lost, this review uncovered opportunities to improve the consultation process; more surgeon involvement and postconsultation survey may be needed.

Although tens of millions of patients worldwide have had successful LASIK, reports of highly dissatisfied patients led to a review by the US Food and Drug Administration [8]. Shortly thereafter, a publication on the peer-reviewed literature from 1988 to 2008 found that >95% of post-LASIK patients were satisfied, which compared as favorably as (or more favorably than) other elective procedures like rhinoplasty, botulinum toxin injections, and breast augmentation [2]. With eye-tracking, improved patient selection, femtosecond laser, and customized ablations, articles from 2008 to 2015 found fewer (1.2%) patients reported dissatisfaction [3].

One paper [9] found that 22% of patients seeking refractive surgery at a university-based center between 2005 and 2010 “changed their mind” after surgical consultation. The main reason was the perception that the risks of surgery outweighed its benefits; the authors noted that this group shrunk over time, possibly because of improved technology and perception of decreased risk. Another paper described cost or “unscientific apprehension” as the reason 15% of patients did not proceed with LASIK [10].

At first glance, our categories may appear arbitrary; one might wonder whether patients who were lost despite good candidacy were possibly the same people whose expectations would not be met. We found these two groups were different in age and type of refractive error: patients whose expectations would not be met had a mean age of 47.4 ± 12.1 years and consisted of more hyperopes, in contrast with lost candidates who were younger (38.3 ± 12 years) and myopic. More than half the patients whose expectations would not be met desired presbyopic correction. Our review showed that a monovision trial was rarely offered; only 9% of surgical patients opted for full or mini-monovision with fewer than half of 40–59-year-old patients (for whom presbyopia was the most common complaint) and 20% of those over age 60 proceeding to surgery.

A substantial proportion (20%) of consultation seekers was lost despite good candidacy and lack of stated reservations about surgery; 58% of lost candidates were low myopes. It is possible, however, that cost, though not stated, was an issue for some “lost candidates.” This is particularly true for ICL and refractive lens exchange, which cost >4 times higher than PRK or LASIK (which cost the same) because of anesthesiologist and surgery center facility fees. Therefore, while some ICL and refractive lens exchange candidates stated they would not proceed because cost and risk were higher than for LASIK, some “lost” candidates might just not have voiced the same concerns. After the recession, our institute reduced the price of excimer laser surgery by 20% with no subsequent volume increase. All patients who undergo refractive surgery consultations, whether determined to be good candidates or not, are reminded of the option of glasses and/or contact lenses; two patients (keratoconus and corneal ectasia, both in the noncandidate category) were referred for the first time for specialty contact lens fitting. Many “lost” candidates likely returned to contact lenses or spectacles.

Hyperopic patients formed the largest proportion of noncandidates and were older than myopes, consistent with the increasing prevalence of hyperopia with age [11]. Hyperopes formed the smallest group of refractive categories seeking consultation, completing surgery, or being lost candidates. Low prevalence alone (9.9% in population-based studies) [11] may limit impetus to expand hyperopic treatment offerings.

Although more women than men sought consultation (possibly related to population-based findings that “severe myopia” (i.e., ≥5 D) is significantly more prevalent in women than in men) [11, 12], male gender was associated with surgery completion. Men were less likely to be noncandidates from a medical or ophthalmic condition like dry eye. Gender differences in communication, in perception of surgery risks, and in vocational needs (e.g., more men than women serve in the military or are first responders) may be other reasons that more men complete surgery.

One might expect economic downturns to affect volume of elective procedures more so than of other surgical procedures because of high out-of-pocket cost and patient selectivity. Surprisingly, the decrease in blepharoplasty, liposuction, rhinoplasty, breast augmentation, and rhytidectomy was less than expected—about the same as the decrease in angioplasty, breast and pancreatic cancer surgery, and hip/knee arthroplasties [13]. It is unclear whether LASIK is purely cosmetic although it compares similarly in-patient satisfaction and quality of life [2]. Hand-held device use, ride-sharing services, and toric and bifocal contact lenses may be of decreasing interest in surgery. Refractive surgeons, however, could spend more effort at patient education; the public may be unaware of advances, base decisions on (negative) lay press [14] or on an outdated consultation, or rely on optometric advice.

Last, the slowdown in refractive surgery volume may have unforeseen consequences. Conclusions drawn from an academic center may not be universally applicable, but such centers train a large fraction of refractive surgeons. Without addressing points raised herein—patient education, outreach, and follow-up—low volume may adversely affect refractive surgery training [15].

## 5. Conclusions

Cost cannot wholly explain the decline in refractive surgery volume; other elective procedures with high out-of-pocket costs are not seeing declines of this magnitude and duration. Our study may lead surgeons to alter current practices regarding education, evaluation, outreach, and follow-up of refractive surgery candidates. For example, more patients might choose monovision rather than forego surgery if they had known about it although monovision alone will not address demand for presbyopic correction. Education (including updating patients on options for treating extreme refractive errors), careful examination and assessment, and communication with the patient even after the visit are key components of a refractive surgery consultation. Such emphasis may generate interest and improve the environment for training future refractive surgeons.

## Figures and Tables

**Table 1:** Age distribution of consultation seekers by gender.

Age in years	Female (% of total seekers)	Male (% of total seekers)	Total
21–39	233 (32.7%)	185 (26%)	418 (58.7%)
40–59	142 (20%)	115 (16.2%)	257 (36.1%)
≥60	26 (3.6%)	11 (1.5%)	37 (5.2%
Total	401 (56.3%)	311 (43.7%)	712 (100%)

## Data Availability

The datasets used and/or analyzed during the current study are available from the corresponding author on reasonable request.

## References

[1] Lindstrom R. L. (2011). Lindstrom’s perspective: continued economic \downturn affecting LASIK, PRK volumes (ocular surgery news). https://www.healio.com/ophthalmology/refractive-surgery/news/print/ocular-surgery-news/%7Ba3543344-f6bc-438c-b49a-705028180fa8%7D/continued-economic-downturn-affecting-lasik-prk-volumes.

[2] Solomon K. D., Fernández de Castro L. E., Sandoval H. P. (2009). LASIK world literature review. *Ophthalmology*.

[3] Sandoval H. P., Donnenfeld E. D., Kohnen T. (2016). Modern laser in situ keratomileusis outcomes. *Journal of Cataract & Refractive Surgery*.

[4] Kuo I. C. (2011). Trends in refractive surgery at an academic center: 2007–2009. *BMC Ophthalmol*.

[5] Sharma N., Singhvi A., Sinha R., Vajpayee R. B. (2005). Reasons for not performing LASIK in refractive surgery candidates. *Journal of Refractive Surgery*.

[6] Hori-Komai Y., Toda I., Asano-Kato N., Tsubota K. (2002). Reasons for not performing refractive surgery. *Journal of Cataract & Refractive Surgery*.

[7] Bamashmus M., Saleh M. F., Abdulrahman M., Al-Kershy N. (2010). Reasons for not performing LASIK in refractive surgery candidates in Yemen. *European Journal of Ophthalmology*.

[8] Latest of FDA’s LASIK Program (Food and Drug Administration website), 2008. https://www.fda.gov/MedicalDevices/ProductsandMedicalProcedures/SurgeryandLifeSupport/ucm061421.htm

[9] Xu K., McKee H. D., Jhanji V. (2013). Changing perspective of reasons for not performing laser-assistedin situkeratomileusis among candidates in a university eye clinic. *Clinical and Experimental Optometry*.

[10] Hashmani S., Hashmani N., Kumar S. (2017). Reasons for refusing laser-assisted in situ keratomileusis in a Pakistani population. *Cureus*.

[11] Kempen J. H., Mitchell P., Lee K. E. (2004). Eye diseases prevalence research group. The prevalence of refractive errors among adults in the United States, western Europe, and Australia. *Archives of Ophthalmology*.

[12] Vitale S., Ellwein L., Cotch M. F., Ferris F. L., Sperduto R. (2008). Prevalence of refractive error in the United States, 1999–2004. *Archives of Ophthalmology*.

[13] Fujihara N., Lark M. E., Fujihara Y., Chung K. C. (2017). The effect of economic downturn on the volume of surgical procedures: a systematic review. *International Journal of Surgery*.

[14] Rabin R. C. (2018). Blurred vision, burning eyes: this is a LASIK success? (New York times). https://www.nytimes.com/2018/06/11/well/lasik-complications-vision.html/.

[15] Lemanski N., Waite A., Kezirian G. M. (2016). Resuscitating refractive surgery (ophthalmology management). https://www.ophthalmologymanagement.com/issues/2016/november-2016/resuscitating-refractive-surgery.

